# Elongator Complex Influences Telomeric Gene Silencing and DNA Damage Response by Its Role in Wobble Uridine tRNA Modification

**DOI:** 10.1371/journal.pgen.1002258

**Published:** 2011-09-01

**Authors:** Changchun Chen, Bo Huang, Mattias Eliasson, Patrik Rydén, Anders S. Byström

**Affiliations:** 1 Department of Molecular Biology, Umeå University, Umeå, Sweden; 2 Division of Epidemiology, Department of Medicine and Public Health, Vanderbilt University School of Medicine, Nashville, Tennessee, United States of America; 3 Department of Chemistry, Umeå University, Umeå, Sweden; 4 Computational Life Science Cluster (CLiC), Umeå University, Umeå, Sweden; 5 Department of Mathematics and Mathematical Statistics, Umeå University, Umeå, Sweden; University of California San Francisco, United States of America

## Abstract

Elongator complex is required for formation of the side chains at position 5 of modified nucleosides 5-carbamoylmethyluridine (ncm^5^U_34_), 5-methoxycarbonylmethyluridine (mcm^5^U_34_), and 5-methoxycarbonylmethyl-2-thiouridine (mcm^5^s^2^U_34_) at wobble position in tRNA. These modified nucleosides are important for efficient decoding during translation. In a recent publication, Elongator complex was implicated to participate in telomeric gene silencing and DNA damage response by interacting with proliferating cell nuclear antigen (PCNA). Here we show that elevated levels of tRNA^Lys^_s^2^__UUU_, tRNA^Gln^_s^2^__UUG_, and tRNA^Glu^_s^2^__UUC_, which in a wild-type background contain the mcm^5^s^2^U nucleoside at position 34, suppress the defects in telomeric gene silencing and DNA damage response observed in the Elongator mutants. We also found that the reported differences in telomeric gene silencing and DNA damage response of various *elp3* alleles correlated with the levels of modified nucleosides at U_34_. Defects in telomeric gene silencing and DNA damage response are also observed in strains with the *tuc2*Δ mutation, which abolish the formation of the 2-thio group of the mcm^5^s^2^U nucleoside in tRNA^Lys^_mcm^5^_s^2^__UUU__, tRNA^Gln^_mcm^5^_s^2^__UUG__, and tRNA^Glu^_mcm^5^_s^2^__UUC__. These observations show that Elongator complex does not directly participate in telomeric gene silencing and DNA damage response, but rather that modified nucleosides at U_34_ are important for efficient expression of gene products involved in these processes. Consistent with this notion, we found that expression of Sir4, a silent information regulator required for assembly of silent chromatin at telomeres, was decreased in the *elp3*Δ mutants.

## Author Summary

Elongator is a conserved protein complex in eukaryotes. Studies in yeast, worms, and plants have revealed that Elongator complex is required for formation of mcm^5^ and ncm^5^ side chains at wobble uridines in a subset of tRNA species. The primary function of Elongator complex in yeast is to modify U_34_ in tRNAs. Lack of these tRNA modifications causes pleiotropic phenotypes in yeast Elongator mutants due to inefficient translation. In this report, we demonstrate that the defects in telomeric silencing and DNA damage response observed in yeast Elongator mutants are a consequence of a tRNA modification defect. We suggest that the requirement of Elongator complex in tRNA modification is conserved in all eukaryotes, and diseases linked to human Elongator mutations may involve impaired translation due to lack of tRNA modifications.

## Introduction

Elongator complex, first identified in *Saccharomyces cerevisiae*, consists of a core complex, Elp1–Elp3 and a sub-complex, Elp4–Elp6 [1]–[3]. Orthologs of Elp1 to Elp4 has been identified in higher eukaryotes and a six-subunit Elongator complex has been purified from humans [4]–[5]. In yeast, Elongator mutants display pleiotropic phenotypes in multiple cellular processes including RNA polymerase II transcription and exocytosis [1]–[3], [6]–[9]. A crucial observation in understanding the role of the yeast Elongator complex was the discovery of its requirement for formation of 5-carbamoylmethyl (ncm^5^) and 5-methoxycarbonylmethyl (mcm^5^) side chains of wobble uridines [10]. In yeast Elongator mutants, the formation of ncm^5^ and mcm^5^ side chains were abolished in the 11 tRNA species that normally contain one of these two side chains [10]–[12]. Elongator complex in *C. elegans* and *A. thaliana* is also required for formation of ncm^5^ and mcm^5^ side chains at wobble uridines [13]–[14]. When the ncm^5^ and mcm^5^ side chains were eliminated, the corresponding tRNA species acted less efficiently in translation [12]. Although lack of modifications at position 5 affects the decoding properties of many tRNAs, it appears that the pleiotropic phenotypes of Elongator mutants are predominantly due to decreased translational decoding by hypomodified 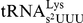 and 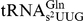 [15]. Simultaneous over-expression of hypomodified 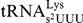 and 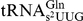, which both have the mcm^5^s^2^U modification at wobble position U_34_ in wild type strains, compensated all phenotypes observed in Elongator mutants including those in RNA polymerase II transcription and exocytosis without restoring formation of ncm^5^ and mcm^5^ side chains in tRNA [15]. These observations not only argue against a direct involvement of Elongator complex in other cellular processes than tRNA modification, but they also suggest that the mcm^5^ side chain is important for efficient translation of mRNAs encoding gene products critical for the processes in which Elongator mutants generate phenotypes.

In eukaryotes, the whole genome is packed into a nucleoprotein complex known as chromatin through which the genetic material is processed to regulate cellular processes including transcription, cell division, DNA replication and DNA repair [16]–[17]. Chromatin properties can be altered by the posttranscriptional modifications of histones including acetylation, methylation, phosphorylation and ubiquitination [16]. The Elp3 protein of Elongator complex contains a tentative histone acetyltransferase (HAT) domain in the C-terminal region and the histone acetylation levels are decreased in *elp3* mutants [7]. However, the reduced histone acetylation levels in the *elp3* mutant were restored by increased expression of 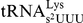 and 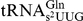, indicating that the involvement of Elongator complex in chromatin remodeling is indirect [15]. In addition to the HAT domain, Elp3 contains an N-terminal region with sequence similarity to the radical S-adenosylmethionine (SAM) enzymes [18]. A recent report showed that Elongator mutants have a partial loss of telomeric gene silencing and are sensitive to DNA damage agents [19]. It was also observed that strains with different point mutations in the *ELP3* gene, resulting in amino acid substitutions in the radical SAM and HAT domains, displayed differences in telomeric gene silencing and DNA damage response [19]. The participation of Elongator complex in telomeric gene silencing and DNA damage response was linked to its interaction with proliferating cell nuclear antigen (PCNA), a protein involved in DNA replication and DNA repair [19].

In this report, we demonstrate that defects observed in DNA damage response and telomeric gene silencing of yeast Elongator mutants are caused by the absence of wobble uridine tRNA modifications. So far, all phenotypes observed in yeast Elongator mutants can be explained by their influence on tRNA modification. We conclude that the primary role of Elongator complex in yeast is in formation of ncm^5^ and mcm^5^ side chains at U_34_ of tRNAs.

## Results

### Elevated levels of hypomodified tRNA^Lys^_s^2^__UUU_, tRNA^Gln^_s^2^__UUG_, and tRNA^Glu^_s^2^__UUC_ suppress defects in telomeric silencing and DNA damage response induced by Elongator mutants

**Figure 1:**
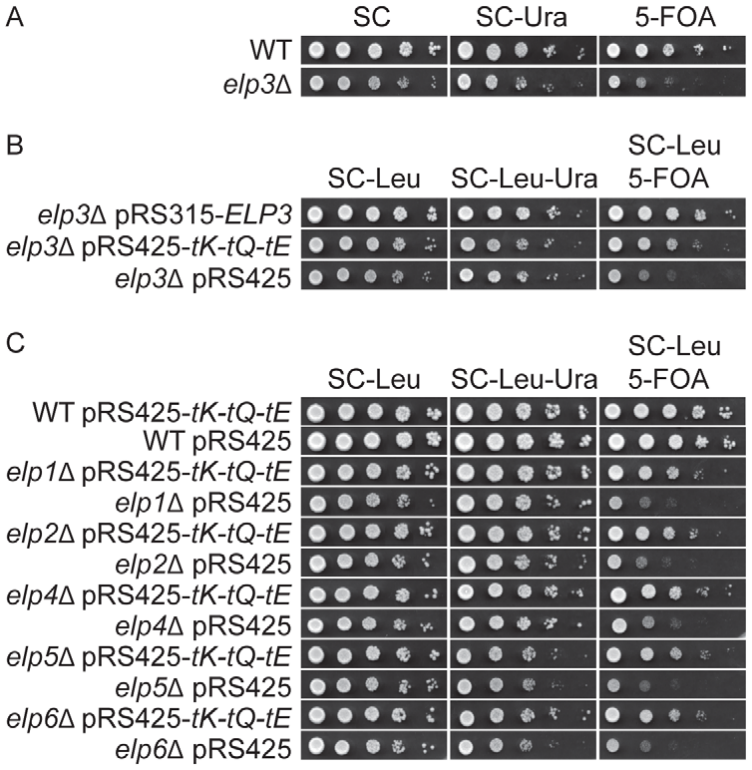
Increased levels of tRNA^Lys^_s^2^__UUU_, tRNA^Gln^_s^2^__UUG_, and tRNA^Glu^_s^2^__UUC_ suppress the telomeric silencing defect of Elongator mutants. (A) The wild type (UMY2584) and *elp3*Δ (UMY3790) strains were 10-fold diluted, spotted on SC, SC-Ura and SC+5-FOA plates, and incubated at 30°C for 2 days. (B) The *elp3*Δ strain (UMY3790) with plasmids, pRS315-*ELP3*, pRS425-*tK-tQ-tE* or pRS425, were 10-fold diluted, spotted on SC-Leu, SC-Leu-Ura and SC-Leu+5-FOA plates, and incubated at 30°C for 2 days. (C) The wild type (UMY2584), *elp1*Δ (UMY3788), *elp2*Δ (UMY3789), *elp4*Δ (UMY3791), *elp5*Δ (UMY3792) and *elp6*Δ (UMY3793) with plasmids pRS425-*tK-tQ-tE* or pRS425 were treated as described in (B). Abbreviations for the tRNA genes encoding 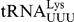, 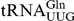 and 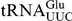 are *tK*, *tQ* and *tE*, respectively.

**Figure 2:**
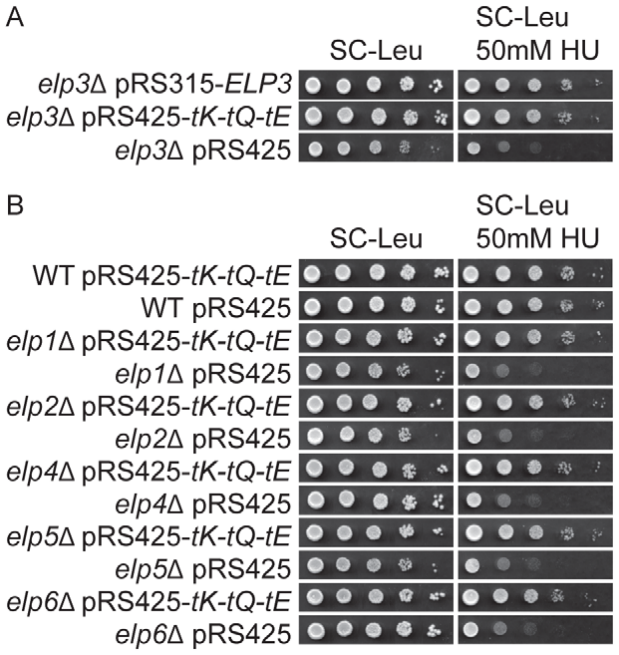
Elevated levels of tRNA^Lys^_s^2^__UUU_, tRNA^Gln^_s^2^__UUG_, and tRNA^Glu^_s^2^__UUC_ suppress the HU sensitivity induced by Elongator mutants. (A) The *elp3*Δ strain (UMY2843) carrying plasmids pRS315-*ELP3*, pRS425-*tK-tQ-tE* or pRS425 were 10-fold diluted, spotted on SC-Leu and SC-Leu+50 mM HU plates, and incubated at 30°C for 2 days. (B) The wild type (W303-1A), *elp1*Δ (UMY3783), *elp2*Δ (UMY3784), *elp4*Δ (UMY3785), *elp5*Δ (UMY3786) and *elp6*Δ (UMY3787) strains transformed with plasmids pRS425-*tK-tQ-tE* or pRS425 were assayed as described in (A). Abbreviations for the tRNA genes encoding tRNA^Lys^_UUU_, tRNA^Gln^_UUG_, and tRNA^Glu^_UUC_ are *tK*, *tQ* and *tE*, respectively.

In a recent report, Elongator mutants were shown to have decreased telomeric gene silencing, which was investigated by using an *ura3-1* strain with a wild-type copy of the *URA3* gene inserted near the left telomere of chromosome VII [19]. Cells with increased expression of Ura3 show reduced growth on plates containing 5-fluoroorotic acid (5-FOA) since the nontoxic 5-FOA is converted to the toxic 5-flurouracil by the *URA3* gene product. In such a strain, 30–50% of the cell population are resistant to 5-FOA [20]. The *URA3* gene was expressed in a population of cells in both wild type and *elp3*Δ strains (Figure 1A). However, the *elp3*Δ strain grew poorly on the 5-FOA containing plates compared to the wild type (Figure 1A), suggesting that telomeric gene silencing was decreased in the *elp3*Δ strain. Since we earlier showed that the primary function of Elongator complex is in formation of wobble uridine tRNA modifications, we investigated whether increased levels of hypomodified 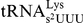, 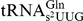 and 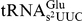 could suppress the defects in telomeric gene silencing of an *elp3*Δ strain. Over-expression of these tRNA species significantly improved the growth of the *elp3*Δ strain on 5-FOA plates (Figure 1B). The telomeric gene silencing defect of Elongator mutants was also investigated by using a color assay with the *ADE2* marker inserted near the telomeric region. The *elp3* mutant forms white color colonies due to loss of silencing of *ADE2*, which could be rescued by increased expression of 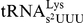, 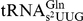 and 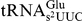 (data not shown). This observation confirmed that Elongator mutants have a defect in telomeric gene silencing, which is caused by a translational dysfunction. The decreased telomeric silencing observed in other Elongator deletion mutants (*elp1*Δ, *elp2*Δ, *elp4*Δ, *elp5*Δ and *elp6*Δ) was also suppressed by elevated levels of 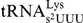, 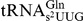 and 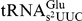 (Figure 1C). Elongator mutants are also sensitive to DNA damaging agents, especially hydroxyurea (HU) [19] (Figure 2). Similar to the defect in telomeric gene silencing, the HU sensitivity of Elongator mutants was suppressed by elevated levels of 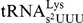, 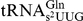 and 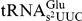 (Figure 2). Collectively, these observations indicate that the reduced gene silencing in telomeric regions and the defect in DNA damage response of Elongator mutants is caused by inefficient translation due to lack of wobble uridine tRNA modifications.

To investigate which of the 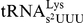, 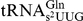 and 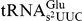 species most efficiently suppressed the defects in telomeric silencing and DNA damage response of the *elp3*Δ strain, we introduced plasmids encoding these tRNAs independently or in various combinations into the mutant. Increased expression of 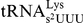 alone could efficiently suppress the telomeric silencing defect and the HU-sensitivity of an *elp3*Δ strain (Figure S1). Simultaneous over-expression of 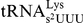, 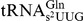 and 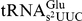 gave a minor improvement in suppression of the telomeric gene silencing defect compared to over-expression of 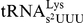 alone (Figure S1A). In the HU sensitivity assay, increased expression of 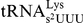 together with 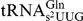 improved the suppression compared to that of 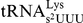 and was as good as elevated levels of 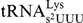, 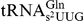 and 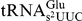 (Figure S1B). These results indicate that certain open reading frames, encoding gene products critical for telomeric gene silencing and DNA damage response, might be enriched in AAA, CAA and GAA codons. Of these three codons, translation of AAA codons by 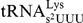 seems to be most affected by lack of the mcm^5^ side chain.

### Synergistic growth reduction and HU sensitivity of *elp3*Δ *asf1*Δ or *elp3*Δ *rtt109*Δ strains are compensated by increased expression of tRNA^Lys^_s^2^__UUU_, tRNA^Gln^_s^2^__UUG_, and tRNA^Glu^_s^2^__UUC_

**Figure 3:**
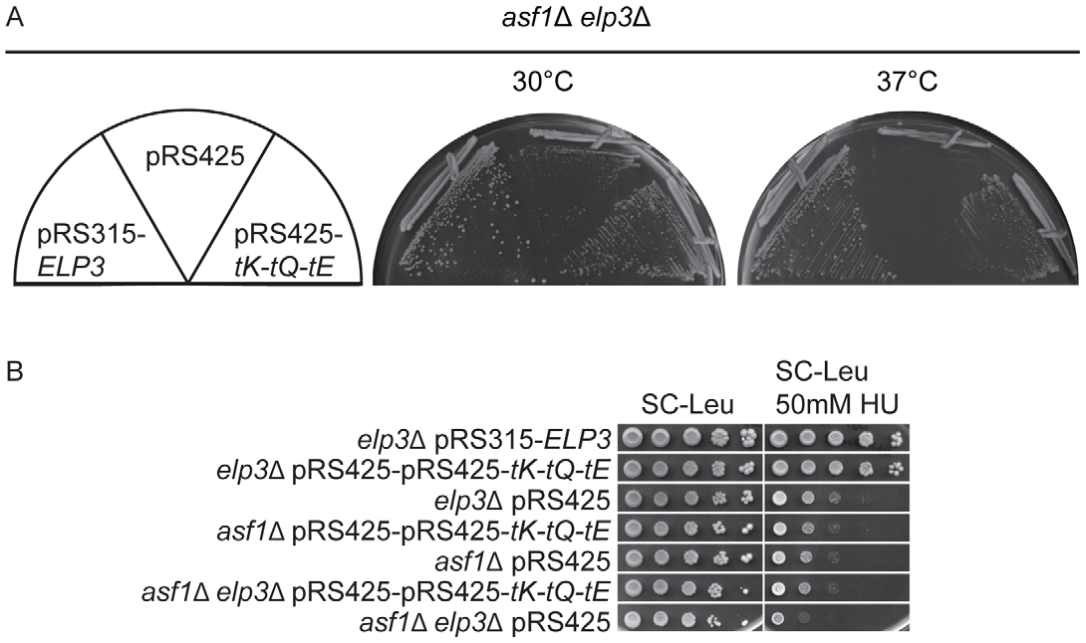
Increased levels of tRNA^Lys^_s^2^__UUU_, tRNA^Gln^_s^2^__UUG_, and tRNA^Glu^_s^2^__UUC_ bypass the phenotypes of *asf1*Δ *elp3*Δ double mutants. (A) The *asf1*Δ *elp3*Δ strain (UMY3805) was transformed with pRS315-*ELP3*, pRS425-*tK-tQ-tE* or pRS425. Transformants were streaked on SC-Leu plates and incubated at 30°C or 37°C for 2 days. (B) Ten fold dilutions of *elp3*Δ (UMY2843), *asf1*Δ (UMY3800) and *asf1*Δ *elp3*Δ (UMY3805) strains carrying either pRS425-*tK-tQ-tE* or pRS425 were spotted on SC-Leu and SC-Leu+50 mM HU plates, and incubated 4 days at 30°C. The *elp3*Δ (UMY2843) transformed with pRS315-*ELP3* was used as control. Abbreviations for the tRNA genes encoding tRNA^Lys^_UUU_, tRNA^Gln^_UUG_, and tRNA^Glu^_UUC_ are *tK*, *tQ* and *tE*, respectively.

Asf1 functions as a histone chaperone to direct the histone acetyltransferase Rtt109 in substrate selection and stimulate its acetyltransferase activity [21]–[23]. The combination of *elp3*Δ *asf1*Δ or *elp3*Δ *rtt109*Δ mutations causes synergistic phenotypes to the strains, such as a more pronounced reduction in growth and increased sensitivity to HU (Figure 3 and Figure S2), which was suggested to be caused by loss of histone acetylation in the *elp3*Δ strain [19]. *GCN5* encodes a histone acetyltransferase that acetylate H2B and H3 [24]–[25]. Previously it was shown that the *elp3*Δ *gcn5*Δ mutations generate a synergistic growth reduction [26]. However, increased levels of hypomodified tRNAs suppressed the synergistic growth reduction caused by the *elp3*Δ *gcn5*Δ mutations, and restore the histone acetylation levels in the *elp3*Δ mutant but not in the *gcn5*Δ strain [15]. When we over-expressed 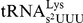, 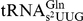 and 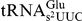 from a high copy vector in the *elp3*Δ *asf1*Δ or *elp3*Δ *rtt109*Δ double mutants, the growth reduction and HU sensitivity of the double mutants were similar to the defects observed in an *asf1*Δ or *rtt109*Δ strain, respectively (Figure 3 and Figure S2). These observations support the earlier conclusion that Elp3 is not directly required for histone acetylation [15].

### Wobble uridine tRNA modification levels correlate to phenotypic variations generated by different mutant alleles of the *ELP3* gene

**Figure 4:**
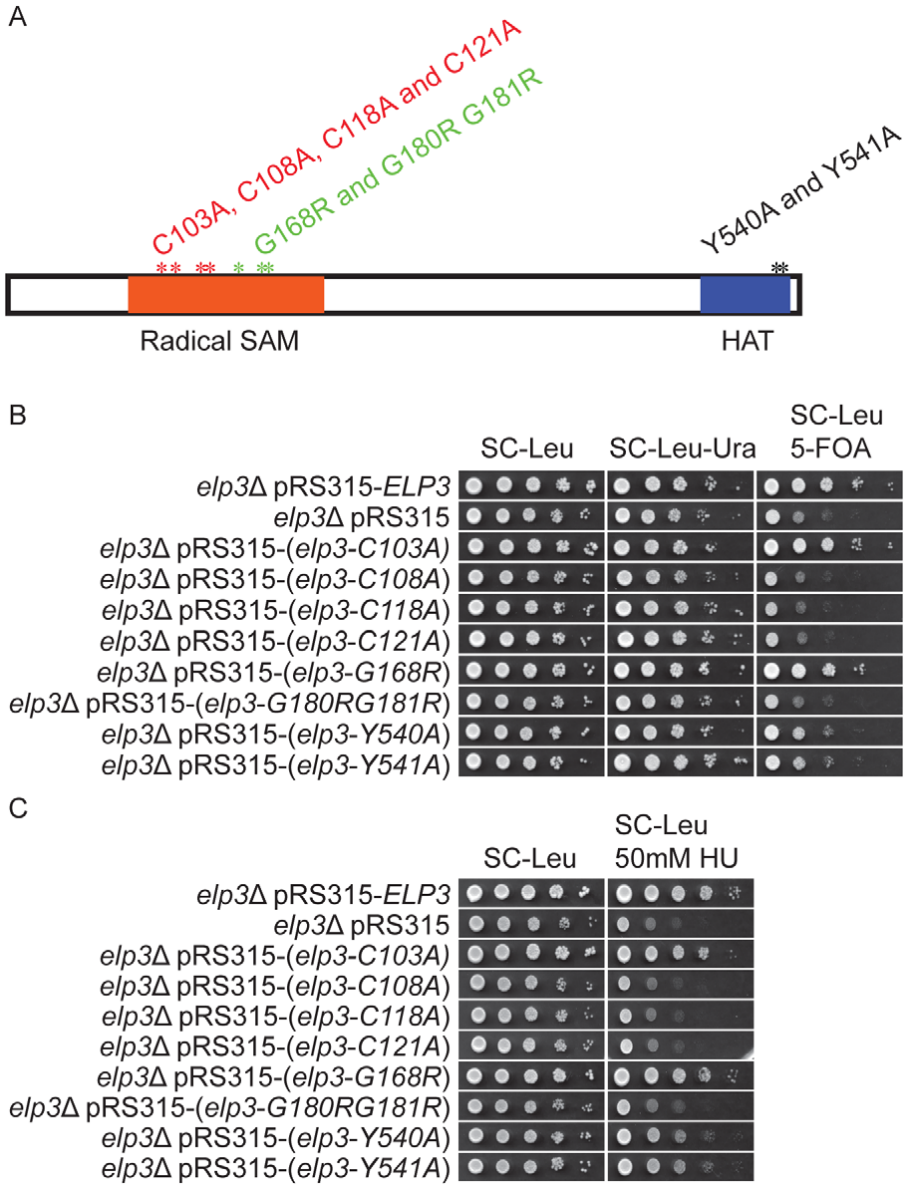
Strains carrying different *ELP3* mutant alleles show decreased telomeric gene silencing and increased HU sensitivity. (A) Schematic drawing of the protein structure of Elp3. Orange box represents the radical-S-adenosyl methionine (Radical-SAM) domain and blue box indicates the location of the histone acetyltransferase (HAT) domain. Cysteine residues at position 103, 108, 118 or 121 were substituted with alanines. Glycine residues at position 168 or 180 together with 181 were replaced by arginines. Two tyrosine residues, positions 540 or 541, in the HAT domain were substituted with alanines. (B) The wild type and the different mutant alleles of the *ELP3* gene, located in *LEU2* containing vector pRS315, were transformed into the *elp3*Δ strain (UMY3790). The *elp3*Δ strain (UMY3790) carrying a pRS315 without insertion serves as control. The transformed yeast cells were spotted on SC-Leu, SC-Leu-Ura and SC-Leu+5-FOA plates, and incubated at 30°C for 2 days. (C) The *elp3*Δ strain (UMY2843) transformed with the same set of plasmids as in (B) were spotted on SC-Leu, SC-Leu+50 mM hydroxyurea plates, and incubated at 30°C for 2 days.

Elp3 contains two conserved domains, a radical S-adenosylmethionine (SAM) domain in the N-terminal region and a putative histone acetyltransferase (HAT) domain located in C-terminal end (Figure 4A). Most strains expressing Elp3 proteins with amino acid substitutions in these two domains showed a reduction in telomeric gene silencing and HU resistance [19] (Figure 4). The *elp3*-*C103A* and *elp3*-*G168R* mutations did not influence telomeric gene silencing and HU sensitivity (Figure 4B and 4C) [19]. The *elp3*-*Y540A* and *elp3*-*Y541A* mutations partially reduced telomeric gene silencing and increased HU sensitivity but not as much as *elp3*Δ (Figure 4B and 4C) [19]. The remaining strains were similar as an *elp3*Δ null strain in telomeric gene silencing and HU sensitivity (Figure 4B and 4C) [19]. Moreover, all strains carrying individual mutations listed in Figure 4A except for *elp3-C103A* were resistant to *Kluyveromyces lactis* killer toxin (data not shown), indicating that these mutants have a defect in formation of wobble uridines tRNA modification [11].

**Figure 5:**
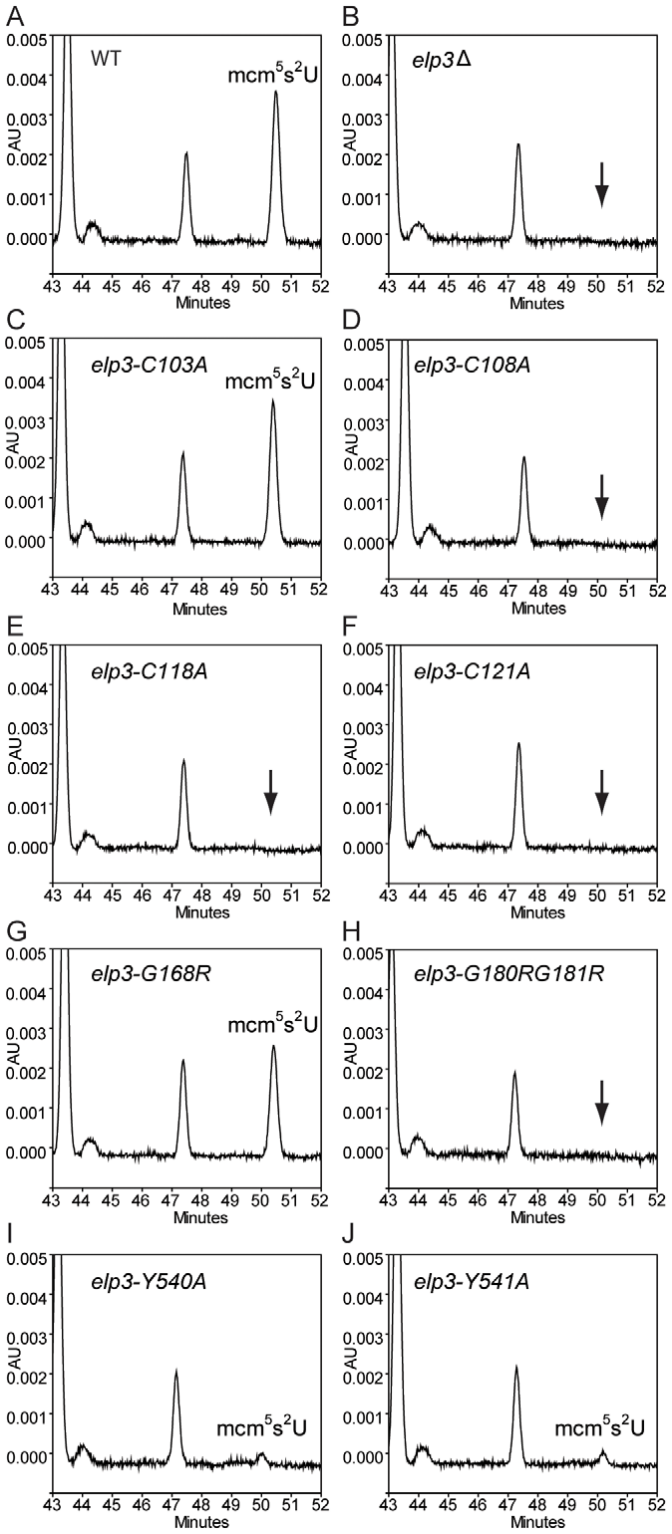
HPLC analysis of total tRNAs isolated from mutants with different alleles of *ELP3*. HPLC chromatograms of total tRNA isolated from *SUP4* (UMY2894), *elp3*Δ *SUP4* (UMY2915), *elp3-C103A SUP4* (UMY3314), *elp3-C108A SUP4* (UMY3315), *elp3-C118A SUP4* (UMY3316), *elp3-C121A SUP4* (UMY3317), *elp3-G168R SUP4* (UMY3794), *elp3-G180R G181R SUP4* (UMY3795), *elp3-Y540A SUP4* (UMY3060) and *elp3-Y541A SUP4* (UMY3061). Chromatograms were monitored at 314 nm. The parts of chromatograms between retention times 43 and 52 min are displayed. The arrows in B, D, E, F and H indicate the expected retention time of mcm^5^s^2^U.

To examine the status of wobble uridine tRNA modification in these *elp3* mutants, total tRNAs from these mutants were isolated and analyzed by HPLC. The *elp3*-*C103A* and *elp3*-*G168R* mutants, which did not have defects in telomeric silencing and DNA damage response, had 96% and 51% mcm^5^s^2^U left, respectively (Figure 5, Table 1). Mutations in the HAT domain did not completely eliminate the formation of wobble uridine modifications, both *elp3*-*Y540A* and *elp3*-*Y541A* have 2 or 6% mcm^5^s^2^U left compared to the wild type (Figure 5, Table 1). In the rest of mutants, the mcm^5^ side chain formation was entirely abolished (Figure 5, Table 1). We conclude that phenotypes exhibited by *elp3* mutants correlate with the levels of wobble uridine tRNA modification.

**Table 1:** Relative amounts of mcm^5^s^2^U analyzed by HPLC in various *elp3* mutants.

Strains	mcm^5^s^2^U/Ψ
*SUP4*	1
*elp3*Δ *SUP4*	ND
*elp3-C103A SUP4*	0.96±0.11
*elp3-C108A SUP4*	ND
*elp3-C118A SUP4*	ND
*elp3-C121A SUP4*	ND
*elp3-G168R SUP4*	0.51±0.08
*elp3-G180R G181R SUP4*	ND
*elp3-Y540A SUP4*	0.018±0.017
*elp3-Y541A SUP4*	0.056±0.015

Pseudouridine (Ψ) was used as an internal standard. The numbers given are the ratios of mcm^5^s^2^U to Ψ in total tRNA isolated from various mutants normalized to the ratio in the wild type *SUP4* strain. Values represent the average of three independent experiments, except for *elp3-Y540A SUP4* and *elp3-Y541A SUP4* that are repeated five times. Standard deviation is shown. ND indicates ‘not detected’. Abbreviations: (Ψ) pseudouridine; and (mcm^5^s^2^U) 5-methoxycarbonylmethyl-uridine.

### Different mcm^5^ modification levels correlate with ochre stop codon read through by a suppressor tRNA

**Figure 6:**
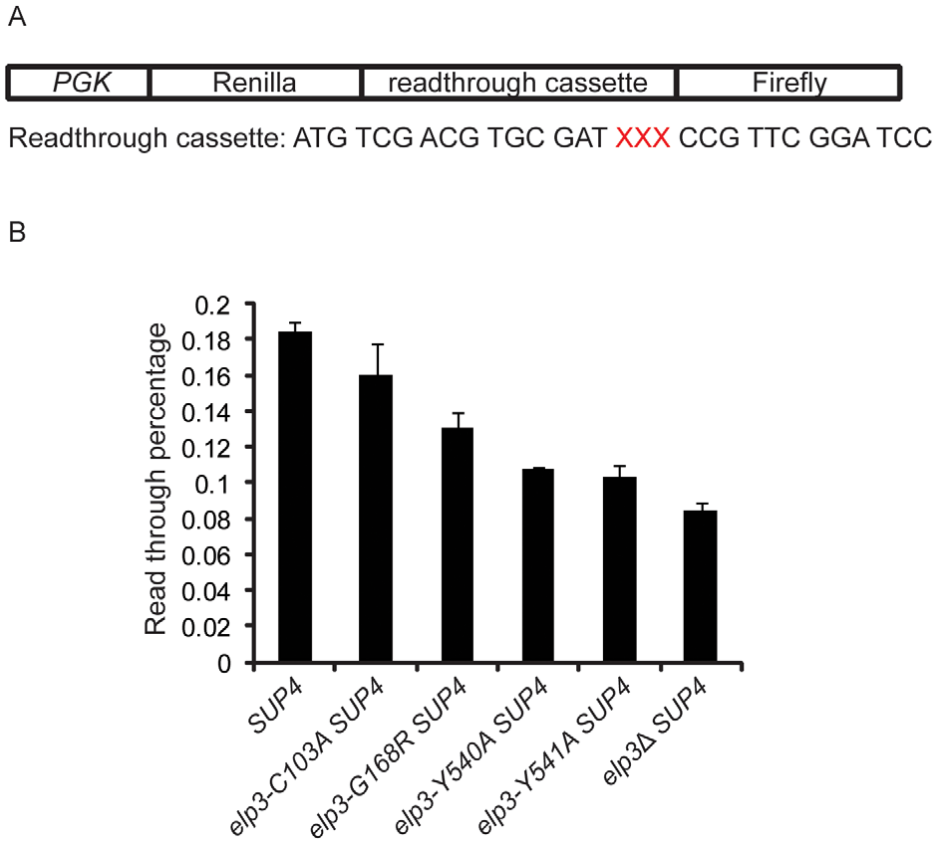
U_34_ modification levels influence ochre stop codon read through by a suppressor tRNA. (A) Schematic drawing of the dual luciferase reporter system constructed by Keeling *et al* [27]. The sequence of the read through cassette between *Renilla* and firefly luciferase genes is shown. The XXX in red stands for either UAA in the assay plasmid or CAA in the control plasmid. (B) Read through levels of the UAA stop codon in *SUP4* (UMY2894), *elp3-C103A SUP4* (UMY3314), *elp3-G168R SUP4* (UMY3794), *elp3-Y541A SUP4* (UMY3060), *elp3-Y541A SUP4* (UMY3061) and *elp3*Δ *SUP4* (UMY2915). Values are ratios of Firefly to Renilla luciferase activities and based on three independent experiments. The error bars represent the standard deviation. Values were normalized to the wild type *SUP4* (UMY2894), which was arbitrarily set to 1.

Our observations suggest that phenotypes of Elongator mutants are caused by an inefficient translation due to lack of tRNA modification. If our model is correct, reduction in modification levels in *elp3* mutants should result in decreased translation efficiency. To analyze whether the modification levels of different *elp3* mutants listed in Table 1 influence translation efficiency, we used a dual-luciferase reporter system (Figure 6A) [27] to measure the ochre stop codon read through by a suppressor tRNA encoded by the *SUP4* allele. The *SUP4* allele encodes a 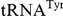 suppressor with a G_34_ to U_34_ substitution in its anticodon. The U_34_ of this suppressor tRNA is modified at position 5 with a mcm side chain [10]. Presence of this modification improves the ability of the suppressor tRNA to read UAA ochre stop codons [10], [12].

In the dual-luciferase construct, the *Renilla* and firefly luciferase genes are separated by an UAA ochre stop codon [27]. Read through of the ochre stop codon was determined by calculating the ratio of firefly luciferase activity to *Renilla* luciferase activity. This ratio was compared to the value obtained from a control construct in which a CAA codon replaces the UAA stop codon (Figure 6A). Due to lack of mcm^5^ side chain in the *SUP4* tRNA, the stop codon read through in the *elp3*Δ strain is reduced to 46% of wild type (*t*-test, p = 0.001), supporting that the mcm^5^ side chain is important for efficient decoding (Figure 6B). In the *elp3*-*G168R* mutant, in which the mcm^5^ side chain is reduced to 51%, the level of read through was significantly decreased compared to that in wild type (*t*-test, p = 0.008), but is higher than that observed in strains carrying the *elp3*-*Y540A*, *elp3*-*Y541A* or *elp3*Δ alleles (*t*-test, p = 0.04 and 0.03 respectively) (Figure 6B). In the *elp3*-*Y540A* and *elp3*-*Y541A* mutants, a small fraction of total tRNA was modified (2–6%) (Figure 5, Table 1), which contributed to an improvement of stop codon read through by the *SUP4* suppressor tRNA compared to the *elp3*Δ strain (*t*-test, p = 0.004 and 0.006 respectively) (Figure 6B). In mutant alleles eliminating formation of the mcm^5^ side chain, no differences were observed in stop codon read through by the *SUP4*-encoded suppressor tRNA compared to the *elp3* null mutant (Figure S3). These data show that reduced mcm^5^ modification levels correlate with decreased translational efficiency.

### Defects in telomeric silencing and DNA damage response are also observed in strains unable to form the s^2^ group of mcm^5^s^2^U

**Figure 7:**
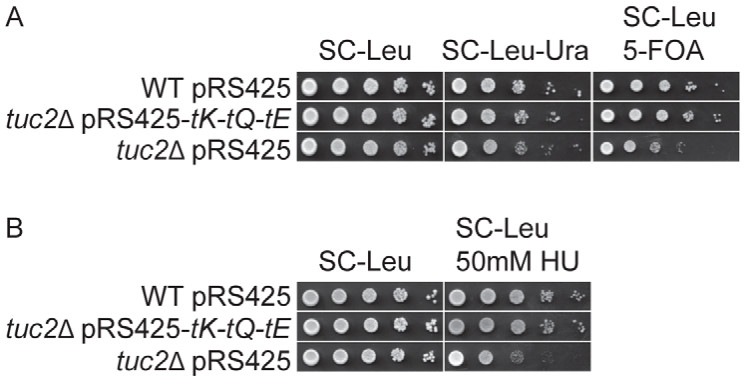
The *tuc2*Δ strain is deficient in telomeric gene silencing and show increased HU sensitivity. (A) The wild type strain (UMY2584) harboring plasmid pRS425 and the *tuc2*Δ mutant (UMY3804) harboring plasmids pRS425-*tK-tQ-tE* or pRS425 were assayed as described in Figure 1. (B) The wild type strain (UMY2067) harboring plasmid pRS425 and the *tuc2*Δ mutant (UMY3442) harboring plasmids pRS425-*tK-tQ-tE* or pRS425 were assayed as described in Figure 2. Abbreviations for the tRNA genes encoding 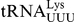, 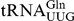 and 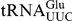 are *tK*, *tQ* and *tE*, respectively.

Our findings that the defects in telomeric silencing and DNA damage response in Elongator mutants were bypassed by elevated levels of 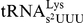, 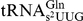 and 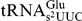 indicated that the mcm^5^ side chain in tRNA is critical for the expression of gene products in these two processes (Figure 1 and Figure 2). In addition to the mcm side chain at position 5 of U_34_, these three tRNAs also contain a 2-thio group forming mcm^5^s^2^U. Since the s^2^ group is also important for decoding [12], [15], [28], we hypothesized that strains deficient in formation of the 2-thio group might also display defects in telomeric silencing and DNA damage response as Elongator mutants. Tuc2 in yeast is required for the formation of the 2-thio group of the mcm^5^s^2^U nucleoside [15]. In a *tuc2*Δ strain, the formation of s^2^ group is abolished. As expected, telomeric gene silencing was decreased in the *tuc2*Δ strain (Figure 7A). This strain was also sensitive to 50 mM HU nearly to the same extent as observed in Elongator mutants (Figure 2 and Figure 7B). The defects in telomeric gene silencing and DNA damage response were completely suppressed by increased levels of 
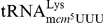
, 
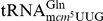
 and 
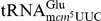
 (Figure 7). The phenotypes of Elongator and *tuc2*Δ mutants demonstrates that a translational dysfunction due to lack of U_34_ modifications in 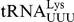, 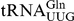 and 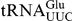 causes the defects in telomeric gene silencing and DNA damage response.

### Sir4 expression is decreased in an *elp3*Δ strain

**Figure 8:**
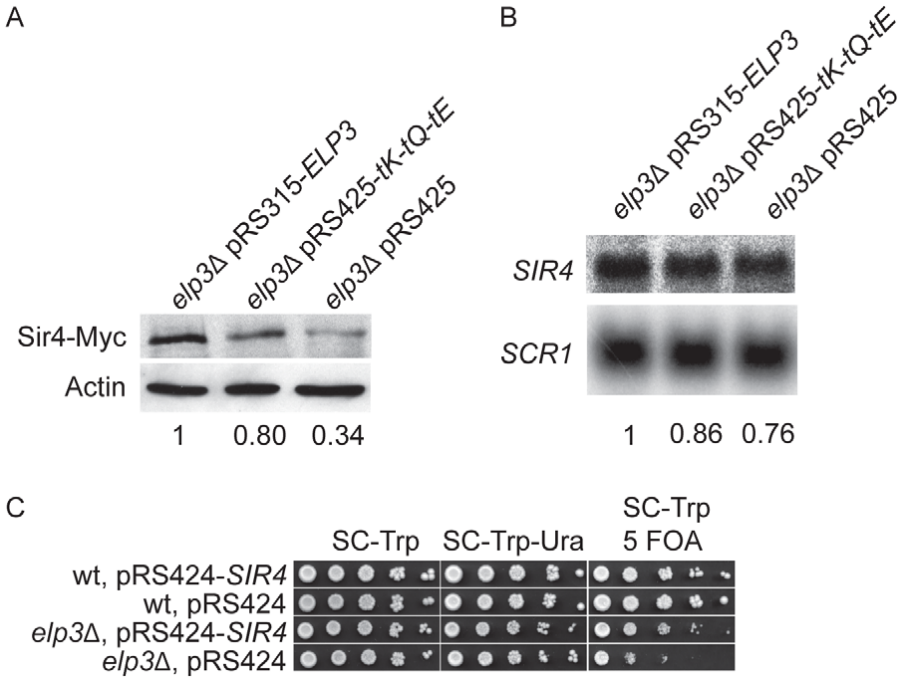
Sir4 protein levels are decreased in the *elp3*Δ mutant. (A). Western blot analysis of Sir4-Myc protein levels in the *elp3*Δ strain transformed with plasmids pRS315-*ELP3*, pRS425-*tK-tQ-tE* or pRS425. The ratios of Sir4-Myc to Actin signals were calculated. The values are shown relative to *elp3*Δ pRS315-*ELP3* strain, which was arbitrarily set to 1, and are the average of two independent experiments. (B). Northern blot analysis of *SIR4* mRNA. The *elp3*Δ strain was transformed with plasmids pRS315-*ELP3*, pRS425-*tK-tQ-tE* or pRS425. Signals of *SIR4* mRNAs were normalized to the non-coding *SCR1* transcript. The values are shown relative to *elp3*Δ pRS315-*ELP3* strain, which was arbitrarily set to 1, and are the average of two independent experiments. (C). The wild type strain (UMY2584) transformed with plasmids pRS424-*SIR4* or pRS424, and the *elp3*Δ mutant (UMY3790) with plasmids pRS424-*SIR4* or pRS424 were assayed as described in Figure 1. For A, B and C, representative figures are shown. Abbreviations for the tRNA genes encoding 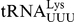, 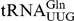 and 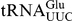 are *tK*, *tQ* and *tE*, respectively.

Among the three tRNA species responsible for the suppression of *elp3*Δ induced phenotypes, increased expression of 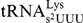 gives the best suppression of the defect in telomeric gene silencing (Figure S1). Since 
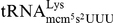
 decodes AAA codons, elimination of the mcm^5^ side chain from 
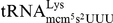
 in the *elp3*Δ strain could influence the decoding efficiency of AAA codons. Therefore, we searched for open reading frames highly enriched in AAA codons (unpublished results). This analysis lead to the identification of *SIR4*, encoding a silent information regulator in yeast. Based on this observation, we hypothesized that the telomeric gene silencing defect of the *elp3*Δ mutant might be caused by decreased Sir4 expression. Accordingly, the Sir4 protein levels in the *elp3*Δ mutant were decreased to 34% of wild type (Figure 8A). The decreased Sir4 levels were restored to 80% of wild-type by increased expression of 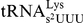, 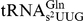 and 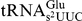, and to 74% of wild-type by elevated levels of 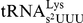 alone (Figure 8A and data not shown). We also observed that *SIR4* mRNA levels were reduced to 76% of wild-type (Figure 8B), which cannot account for the decreased Sir4 protein levels. In addition, introducing the *SIR4* gene on a high copy vector significantly suppressed the telomeric gene silencing defect of the *elp3*Δ strain, confirming that this defect seems to be caused by decreased Sir4 expression (Figure 8C). However, we do not exclude the possibility that there might be other open reading frames enriched in AAA codons whose translation is also affected and which might weaken silencing, directly or indirectly.

## Discussion

Elongator complex was initially identified by its apparent association with the elongating form of RNA polymerase II, implicating a role in PolII transcription [1]. However, its requirement in transcription was controversial based on its cytoplasmic localization and failure to detect this complex on actively transcribed genes [8], [29]–[30]. We discovered that Elongator complex was required for formation of mcm^5^ and ncm^5^ side chains at wobble uridines of tRNA [10]. The participation of Elongator complex in PolII transcription and exocytosis was indirect as elevated expression of hypomodified 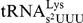 and 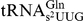 could suppress previously reported phenotypes of Elongator mutants without restoring tRNA modification [15]. Recently, it was reported that Elongator complex modulates telomeric gene silencing and DNA damage response by its interaction with PCNA and its requirement for histone acetylation [19]. Since the histone acetylation defect of the *elp3*Δ mutant could be completely suppressed by increased expression of 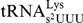 and 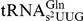 [15], we assumed that Elongator complex indirectly participated in telomeric gene silencing and DNA damage response.

In this report, we show that the defects in telomeric gene silencing and DNA damage response in Elongator mutants were also suppressed by increased expression of hypomodified 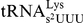, 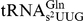 and 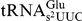 (Figure 1, Figure 2, and Figure S1). Thus, all phenotypes exhibited by Elongator mutants except the tRNA modification defect are overcome by elevated tRNA levels, indicating that the major function of this complex, at least in yeast, is in the formation of mcm^5^ and ncm^5^ side chains of wobble uridines. When 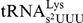, 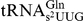 and 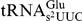 were over-expressed in Elongator mutants, the HU sensitivity phenotype, but not the defect in telomeric gene silencing, was fully suppressed (Figure 1 and Figure 2). Since Elongator mutants affect the mcm^5^ and ncm^5^ side chain formation in 11 tRNA species, it is possible that poor translation of codons decoded by any of the other 8 hypo-modified tRNA species contributes to the defect in telomeric gene silencing, but not the HU sensitivity. In addition to the mcm side chain at position 5, U_34_ of 
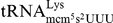
, 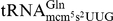 and 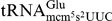 are also thiolated at position 2. If our model is correct that the phenotypes observed in Elongator mutants are a consequence of inefficient translation, strains lacking the 2-thio group in 
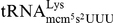
, 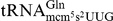 and 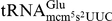 will have similar phenotypes as Elongator mutants. We observed that the failure to form the 2-thio group in the *tuc2*Δ mutant resulted in defects in telomeric gene silencing and DNA damage response (Figure 7). These defects of the *tuc2*Δ mutant were completely suppressed by increased expression of 
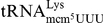
, 
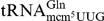
 and 
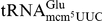
. In addition, lack of the methyl ester in mcm^5^ side chain at wobble uridines in a *trm9*Δ strain has been linked to the defect of DNA damage response [31]. Thus, both mcm^5^ and s^2^ side chains of mcm^5^s^2^U containing tRNAs are important for efficient expression of gene products required for telomeric gene silencing and DNA damage response. These observations strongly suggest that Elongator complex influence these two processes by promoting efficient translation. Since increased expression of 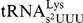 gives the best suppression of the telomeric gene silencing defect in Elongator mutants, we assumed genes encoding products important for this process are enriched in AAA codons. One such gene is *SIR4*. We demonstrate that Elongator mutants influence telomeric gene silencing by impairing efficient expression of *SIR4*. Even though we observed a slight reduction in *SIR4* mRNA levels in the *elp3*Δ mutant, it cannot fully explain the decrease in Sir4 protein levels, and it is unclear if this reduction is caused by reduced transcription or increased decay of the poorly translated mRNA.

Recently, it was discovered that Elongator complex in *C. elegans* and *A. thaliana* is also required for formation of mcm^5^ and ncm^5^ side chains at wobble uridines of tRNA [13]–[14], indicating that this function of Elongator complex might be conserved in eukaryotes. In multicellular organisms, Elongator complex has also been linked to multiple processes including transcription, cytoplasmic kinase signaling and development [32]–[34]. Two recent articles suggested that Elongator complex was also required for α-tubulin acetylation and played a role in neurological processes in both mice and *C. elegans* [35]–[36]. In early developmental stages, *C. elegans* Elongator mutants have a decreased α-tubulin acetylation [36]. However, in adult Elongator mutant worms, normal levels of α-tubulin acetylation were observed, suggesting that Elongator complex is not absolutely required for acetylation of α-tubulin [13], [36]. Elongator mutants in *C. elegans* were also resistant to the acetylcholinesterase inhibitor aldicarb, indicating a reduced efficiency of synaptic exocytosis [13], [36]. However, a mutant allele of *mec-12*, which is completely missing α-tubulin acetylation, was not resistant to aldicarb, suggesting that the defect in synaptic exocytosis of Elongator mutants was not caused by reduced levels of α-tubulin acetylation [13]. Furthermore, *mec-17* was discovered to be the α-tubulin acetylase in in *Tetrahymena* cells, *C. elegans*, zebrafish and mammalian cells, suggesting that Elongator might indirectly influence α-tubulin acetylation by modulating the expression of α-tubulin acetylase [37]. Based on these observations, it is tempting to speculate that the primary function of Elongator complex in multicellular organism is, as in yeast, in formation of wobble uridine tRNA modifications.

The Elp3 subunit in yeast has an N-terminal radical S-adenosylmethionine (SAM) domain and a C-terminal histone acetyltransferase (HAT) domain. In *Methanocaldococcus jannaschii*, the radical SAM domain of mjElp3 contains an iron sulfur cluster region and a region that binds SAM [38]. Cysteine residues at positions 96, 101 and 104 are critical for the FeS cluster formation in *M. jannaschii* [38]. When these corresponding cysteines at position 108, 118 and 121 in the yeast Elp3 were substituted with alanines, it eliminated the activity of yeast Elongator in formation of modified nucleosides at U_34_. *In vitro*, SAM can bind to *M. jannaschii* Elp3, but the binding of SAM to Elp3 from *S. cerevisiae* has not been detected [38]–[39]. However, when the conserved SAM binding sites (G180R G181R) in the radical SAM domain were mutated in yeast *ELP3*, a defect in formation of modified nucleosides was observed (Figure 5, Table 1). This observation shows that the FeS cluster and the SAM binding regions of the radical SAM domain of Elp3 are critical for the tRNA modification reaction. Substitution of glycine at position 168 to arginine, another conserved site located in the SAM binding region, reduced the wobble uridine tRNA modification to 51% of wild type (Figure 5, Table 1). In telomeric gene silencing and HU sensitivity assays, the *elp3-G168R* mutant displays the same phenotypes as a wild type strain suggesting that a 49% reduction in the levels of modified nucleosides do not cause phenotypes in telomeric gene silencing and DNA damage response. Two mutations in the HAT domain (Y540A and Y541A) of Elp3 did not entirely eliminate the formation of modified nucleosides at U_34_; 2 and 6% of mcm^5^s^2^U was detected in each mutant (Table 1). The residual level of modified nucleosides significantly improves the decoding capacity of the *SUP4* encoded suppressor tRNA compared to the unmodified tRNA in the *elp3* null mutant (Figure 6). This observation explains why the *elp3-Y540A* and *elp3-Y541A* mutants had increased telomeric silencing and reduced HU sensitivity compared to the *elp3*Δ strain (Figure 4).

Among the *elp3* mutants described in Table 1, the *elp3-G168R* mutant, having 51% of modified nucleoside left (Figure 5 and Table 1), has the same phenotype as a wild type strain with respect to phenotypes in telomeric gene silencing and DNA damage response (Figure 4). However, this strain is resistant to killer toxin (data not shown), a phenotype tightly connected to wobble uridine tRNA modification [11]. The γ subunit of killer toxin is a tRNA endonuclease which cleaves tRNA at the anticodon region [11]. The mcm^5^ side chain at U_34_ of tRNA is important for the substrate recognition by γ toxin. In the *elp3-G168R* mutant, a fraction of the U_34_ tRNAs are missing the mcm^5^ side chain and the mutant is resistant to γ toxin (data not shown). However, the modified tRNAs in the *elp3-G168R* support the efficient expression of gene products required for telomeric gene silencing and DNA damage response. Thus, strains with tRNAs partially modified at U_34_ show weaker or no phenotypes compared to Elongator deficient strains.

In summary, the major function of Elongator complex in yeast is in formation of wobble uridine tRNA modifications and this function is probably conserved in eukaryotes. We suggest that when new phenotypes of Elongator mutants are discovered in yeast, an important first step is to investigate whether the phenotypes can be suppressed by over-expressing 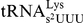, 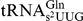 and 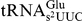.

## Materials and Methods

### Yeast strains, media, and genetic procedures

All yeast strains used in this study are listed in Table S1. Yeast transformation, media, and genetic procedures have been described previously [40]. To generate *elp* null mutants in different strain backgrounds, chromosomal DNA from *KanMX* deleted *elp* mutants UMY2911 (*elp1::KanMX4*), UMY2913 (*elp2::KanMX4*), UMY2915 (*elp3::KanMX4*), UMY2917 (*elp4::KanMX4*), UMY2919 (*elp5::KanMX6*) and UMY2921 (*elp6::KanMX4*) served as templates. Primers were designed to amplify DNA fragments containing the *KanMX* cassette and 300–500 nt flanking sequences of each *ELP* gene. PCR products were transformed into either W303-1A or UMY2584, and the transformants were selected by using YEPD plates containing 200 µg/ml G418. The deletion mutants were verified by PCR. To introduce *asf1::KanMX4* and *rtt109::KanMX4* into W303 background, chromosomal DNAs from the corresponding mutants in the deletion collection (Open biosystems) were used as templates. Primers were designed to amplify the *KanMX4* cassette and 500 nt flanking sequences. PCR products were transformed into diploid strain UMY3104 and transformants were selected on G418 containing plates. The *asf1::KanMX4* and *rtt109::KanMX4* strains were obtained by tetrad dissection after sporulation. To construct *asf1::KanMX4 elp3::KanMX4* and *rtt109::KanMX4 elp3::KanMX4*, the *elp3::KanMX4* strain was crossed with *asf1::KanMX4* or *rtt109::KanMX4* to generate the diploid and double mutants were obtained by tetrad dissection. To generate *elp3::KanMX4 SIR4-13Myc-KanMX6* strain, the *elp3::KanMX4* strain was crossed with *SIR4-13Myc-KanMX6* strain. The diploid was sporulated and the *elp3::KanMX4 SIR4-13Myc-KanMX6* strain was obtained by tetrad dissection.

A two-step gene replacement procedure was used to obtain strains with different mutant alleles of *ELP3*. Plasmids pABY1672 (*elp3-C103A*), pABY1673 (*elp3-C108A*), pABY1676 (*elp3-C118A*), pABY1677 (*elp3-C121A*), pABY1984 (*elp3-G168R*) and pABY1985 (*elp3-G180R G181R*) were digested with *Eco*RI and the linearized fragments were transformed into the UMY2894. Transformants were selected on SC-Ura plates and streaked on YEPD plates. Eight independent colonies on YEPD plates were picked and streaked on 5-FOA containing plates. The strains with *elp3* mutant alleles except for *elp3-C103A* were identified by their resistance to killer toxin and confirmed by sequencing. In order to identify the *elp3-C103A* mutant, DNA isolated from several candidates were sequenced.

### Plasmid constructions

Plasmids used in this study are listed in Table S2. The pRS306-*ELP3* (pABY1554) was constructed previously [10] and used as DNA template for mutagenesis. Plasmids pABY1672 (*elp3-C103A*), pABY1673 (*elp3-C108A*), pABY1676 (*elp3-C118A*), pABY1677 (*elp3-C121A*), pABY1984 (*elp3-G168R*) and pABY1985 (*elp3-G180R G181R*) were generated by using Quickchange Lightning Multi Site-Directed mutagenesis kit according to the instruction manual (Agilent Technologies). Site-specific primers were designed by Agilent online service. To move mutant alleles of *ELP3* to pRS315, pRS306-*elp3* derivatives were digested using restriction enzymes *Bam*HI and *Xho*I, and the excised fragments were cloned into the corresponding sites of pRS315. To generate pRS424-*SIR4*, *SIR4* gene was amplified by PCR using W303-1A genomic DNA as template with oligos AAAA GAATTC TGTGA GTACATATAT CCGCAG and AAAA CTCGAG TTG GTATTTGATG GGTTGCTC. The PCR product was digested with *Eco*RI and *Xho*I, and cloned to the corresponding sites on pRS424.

### tRNA isolation and HPLC analysis

Cells were grown at 30°C in 100 ml YEPD and harvested at OD600 = 1.5∼2. The cell pellet was resuspended in 3 ml 0.9% NaCl. The cell suspension was vortexed at room temperature for 30 minutes in the presence of 8 ml water-saturated phenol and vortexed for another 15 minutes after adding 0.4 ml chloroform. Centrifugation was carried out at 12000 g for 20 minutes. The water phase was collected and re-extracted with phenol. The final water phase was collected, mixed with 2.5 volume 99.5% ethanol and kept at −20°C for at least 3 hours. Total RNA was pelleted at 12000 g for 20 minutes. The RNA pellet was dissolved in 5 ml DE52 binding buffer (0.1 M Tris.HCl pH 7.4 and 0.1 M NaCl) and loaded onto the DE52 cellulose column. The column was washed twice with 7 ml DE52 binding buffer and the tRNA was eluted with 7 ml elution buffer (0.1 M Tris.HCl pH 7.4 and 1 M NaCl). The tRNA was precipitated with 0.7 volume of isopropanol at −20°C for at least 3 hours and pelleted by centrifugation at 12000 g for 20 minutes. The pellet was washed once with 70% ethanol and dissolved in 50 µl MQ. Purified tRNA was digested with Nuclease P1 for 16 hrs at 37°C and treated with bacterial alkaline phosphatase for 2 hours at 37°C. The hydrolysate was analyzed by high pressure liquid chromatography with a Develosil C-30 reverse-phase column as described [41].

### Telomeric gene silencing and DNA damage response assays

To investigate the defect in telomeric gene silencing of Elongator mutants, 10-fold dilutions of freshly cultivated yeast cells were spotted on 5-FOA containing plates and control plates. Plates were incubated at 30°C for 2 days. To analyze the DNA damage response, 10 fold dilutions of freshly cultivated yeast cells were spotted on the plates containing 50 mM HU and control plates. The results were scored after 2 days of incubation at 30°C.

### Dual-luciferase reporter assay

The luciferase activities were measured by GloMax 20/20 luminometer (Promega) and the dual-luciferase reporter assay system (Promega). Cells were grown to 0.5 OD_600_ and diluted 10 fold before use. 20 µl of diluted cell culture was mixed with 100 µl passive lysis buffer, vortexed for 12 seconds and 20 µl of cell lysate was used to determine the luciferase activity. Each culture was measured 3 times and 3 independent experiments were performed.

### Western and Northern blotting

To determine the Sir4 protein levels, cells were grown at 30°C to OD_600_ = 0.5 before harvest. Cells were broken in breaking buffer (40 mM Hepes pH 7.3, 50 mM NH_4_Ac, 10 mM MgCl_2_ and 1 mM DTT) containing Complete Protease Inhibitor Cocktail Tablets (Roche Applied Science) by using FastPrep-24 homogenizer (MP biomedicals). 60 µg proteins were loaded in each lane. Mouse anti-Myc antibody (9E10) with a dilution 1∶1000 was used to detect recombinant proteins. The actin levels, used as an internal control, were detected using mouse anti-Act1 antibody (Thermo Scientific) at a 1∶2000 dilution. RNA levels were determined as previously described [42].

## Supporting Information

Figure S1Telomeric silencing defects and HU sensitivity of *elp3*Δ strains are predominantly suppressed by over-expressing tRNA^Lys^_s^2^__UUU_. (A). The *elp3*Δ strain (UMY3790) with the plasmids pRS315-*ELP3*, pRS425-*tK-tQ-tE*, pRS425-*tK-tQ*, pRS425-*tK-tE*, pRS425-*tQ-tE*, pRS425-*tK*, pRS425-*tQ*, pRS425-*tE* or pRS425 were 10-fold diluted, spotted on SC-Leu, SC-Leu-Ura and SC-Leu+5-FOA plates, and incubated at 30°C for 2 days. (B). The *elp3*Δ strain (UMY2843) transformed with the same set of plasmids as in (A) were 10 fold diluted, and spotted on SC-Leu and SC-Leu+50 mM HU plates. The plates were incubated 2 days at 30°C. Abbreviations for the tRNA genes encoding 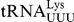, 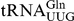 and 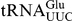 are *tK*, *tQ* and *tE*, respectively.(TIF)

Figure S2Increased levels of tRNA^Lys^_s^2^__UUU_, tRNA^Gln^_s^2^__UUG_, and tRNA^Glu^_s^2^__UUC_ suppress the phenotypes of *rtt109*Δ *elp3*Δ double mutants. (A) The *rtt109*Δ *elp3*Δ strain (UMY3807) carrying pRS315-*ELP3*, pRS425-*tK-tQ-tE* or pRS425 were streaked on SC-Leu plates and incubated at 30°C or 37°C for 2 days. (B). Strains *elp3*Δ (UMY2843), *rtt109*Δ (UMY3798) and *rtt109*Δ *elp3*Δ (UMY3807) were transformed with either pRS425-*tK-tQ-tE* or pRS425, 10 fold diluted and spotted on SC-Leu and SC-Leu+50 mM HU plates. The results were documented after 4 days of incubation at 30°C. The *elp3*Δ strain (UMY2843) transformed with pRS315-*ELP3* was used as control. Abbreviations for the tRNA genes encoding 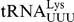, 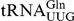 and 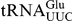 are *tK*, *tQ* and *tE*, respectively.(TIF)

Figure S3 UAA stop codon read through by Sup4 tRNA in strains with different alleles of *elp3*. Read through levels of UAA stop codon in *SUP4* (UMY2894), *elp3-C108A SUP4* (UMY3315), *elp3-C118A SUP4* (UMY3316), *elp3-C121A SUP4* (UMY3317), *elp3-G180R G181R SUP4* (UMY3795) and *elp3*Δ *SUP4* (UMY2915). Values were based on three independent experiments. The error bars represent the standard deviation. The value of *SUP4* (UMY2894) was arbitrarily set to 1 and the others were normalized to UMY2894. The dual luciferase reporter system used for UAA stop codon read through [27] is described in Figure 6A.
(TIF)

Table S1 Yeast strains used in this study (see also [10], [12], [15], [43]).
(DOC)

Table S2 Plasmids used in this study (see also [10]–[11], [27], [44]–[45]).
(DOC)
