# Supplementary figures and images for: Elongator Complex Influences Telomeric Gene Silencing and DNA Damage Response by Its Role in Wobble Uridine tRNA Modification
Source: PLoS Genet. 2011 Sep 1;7(9):e1002258. doi: 10.1371/journal.pgen.1002258 (PMC3164696; doi:10.1371/journal.pgen.1002258)

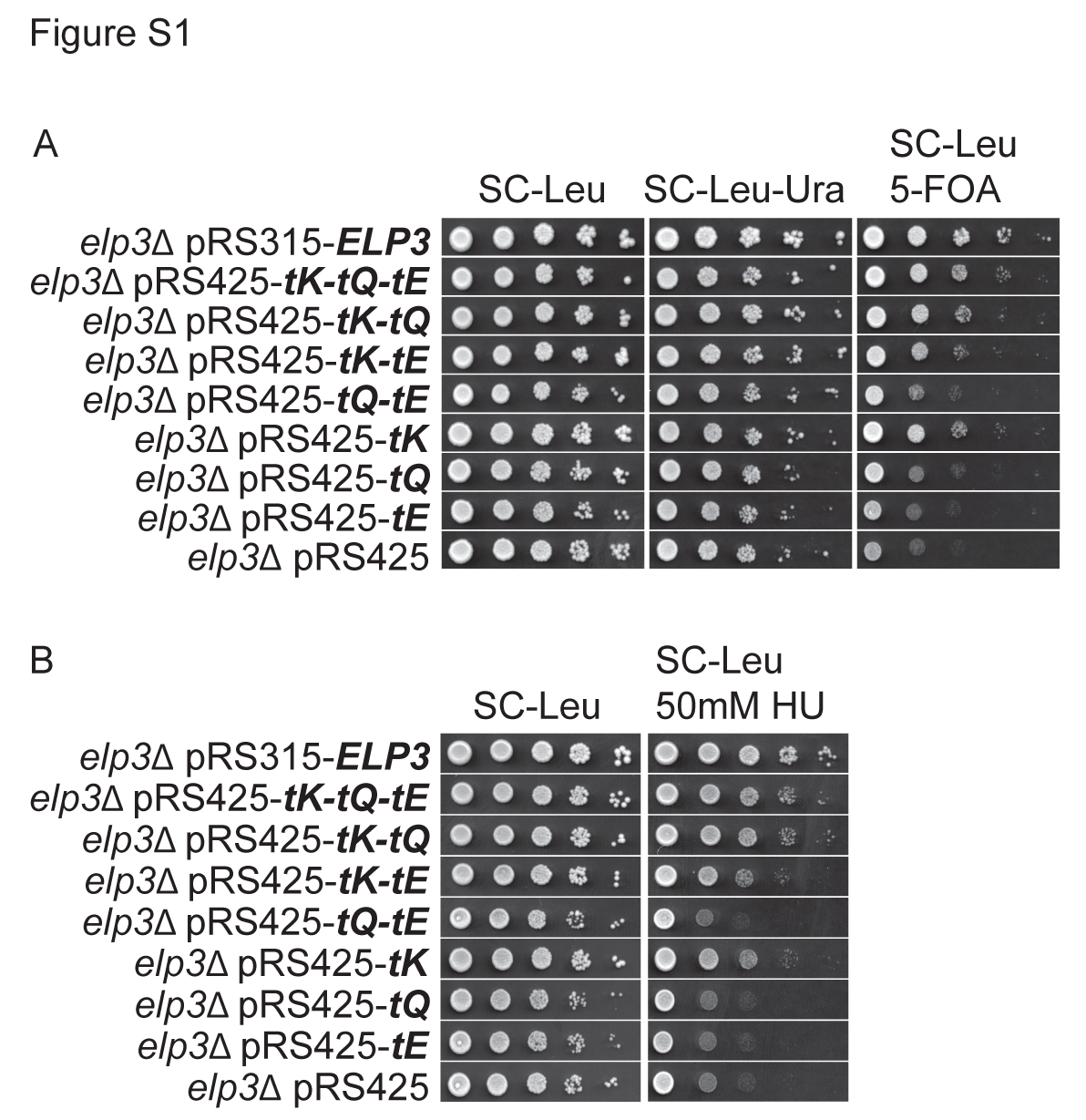

Supplement: Figure S1 — Telomeric silencing defects and HU sensitivity of elp3Δ strains are predominantly suppressed by over-expressing tRNALys s2 UUU. (A). The elp3Δ strain (UMY3790) with the plasmids pRS315-ELP3, pRS425-tK-tQ-tE, pRS425-tK-tQ, pRS425-tK-tE, pRS425-tQ-tE, pRS425-tK, pRS425-tQ, pRS425-tE or pRS425 were 10-fold diluted, spotted on SC-Leu, SC-Leu-Ura and SC-Leu+5-FOA plates, and incubated at 30°C for 2 days. (B). The elp3Δ strain (UMY2843) transformed with the same set of plasmids as in (A) were 10 fold diluted, and spotted on SC-Leu and SC-Leu+50 mM HU plates. The plates were incubated 2 days at 30°C. Abbreviations for the tRNA genes encoding , and are tK, tQ and tE, respectively. (TIF) [file pgen.1002258.s001.tif]

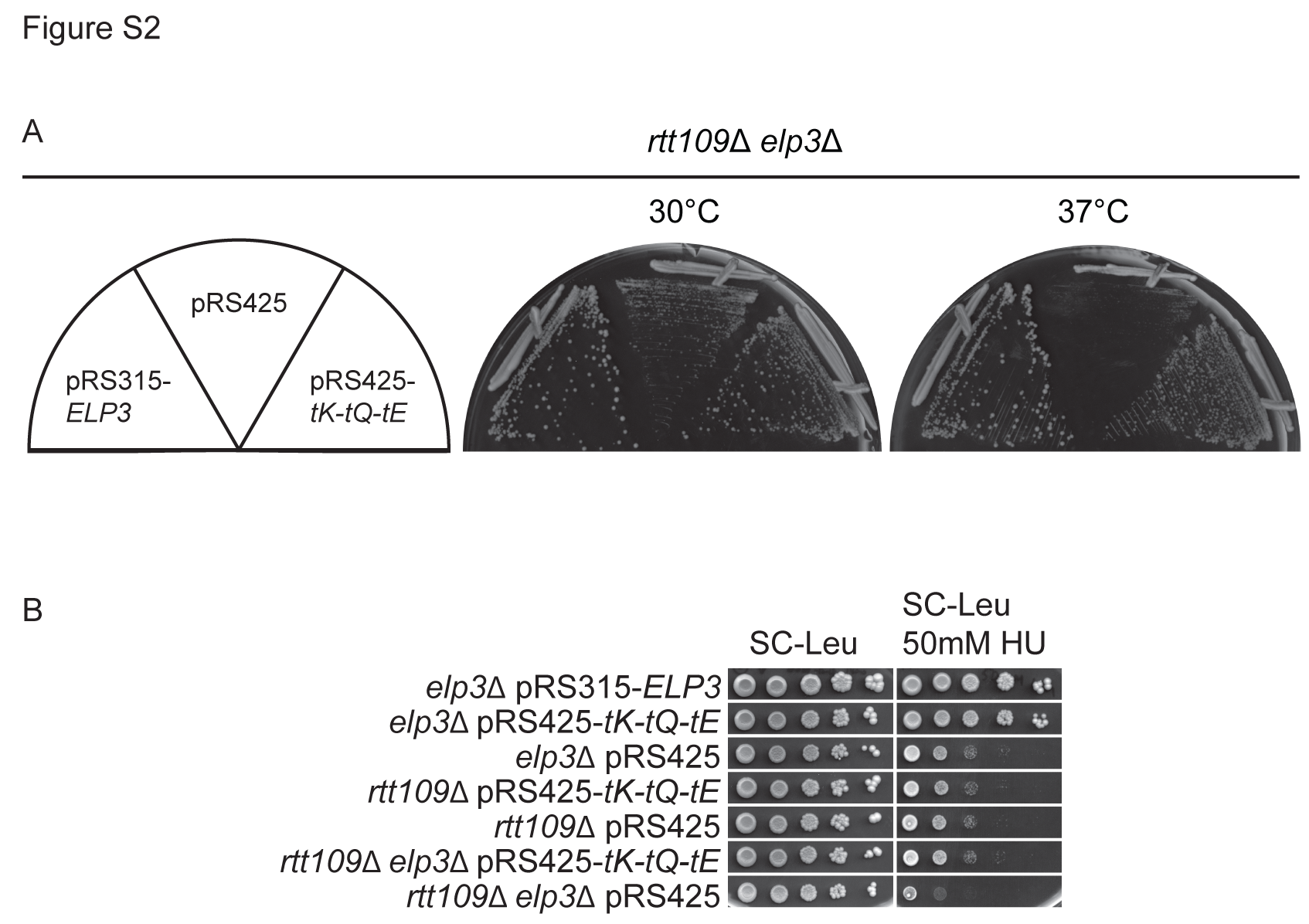

Supplement: Figure S2 — Increased levels of tRNALys s2 UUU, tRNAGln s2 UUG, and tRNAGlu s2 UUC suppress the phenotypes of rtt109Δ elp3Δ double mutants. (A) The rtt109Δ elp3Δ strain (UMY3807) carrying pRS315-ELP3, pRS425-tK-tQ-tE or pRS425 were streaked on SC-Leu plates and incubated at 30°C or 37°C for 2 days. (B). Strains elp3Δ (UMY2843), rtt109Δ (UMY3798) and rtt109Δ elp3Δ (UMY3807) were transformed with either pRS425-tK-tQ-tE or pRS425, 10 fold diluted and spotted on SC-Leu and SC-Leu+50 mM HU plates. The results were documented after 4 days of incubation at 30°C. The elp3Δ strain (UMY2843) transformed with pRS315-ELP3 was used as control. Abbreviations for the tRNA genes encoding , and are tK, tQ and tE, respectively. (TIF) [file pgen.1002258.s002.tif]

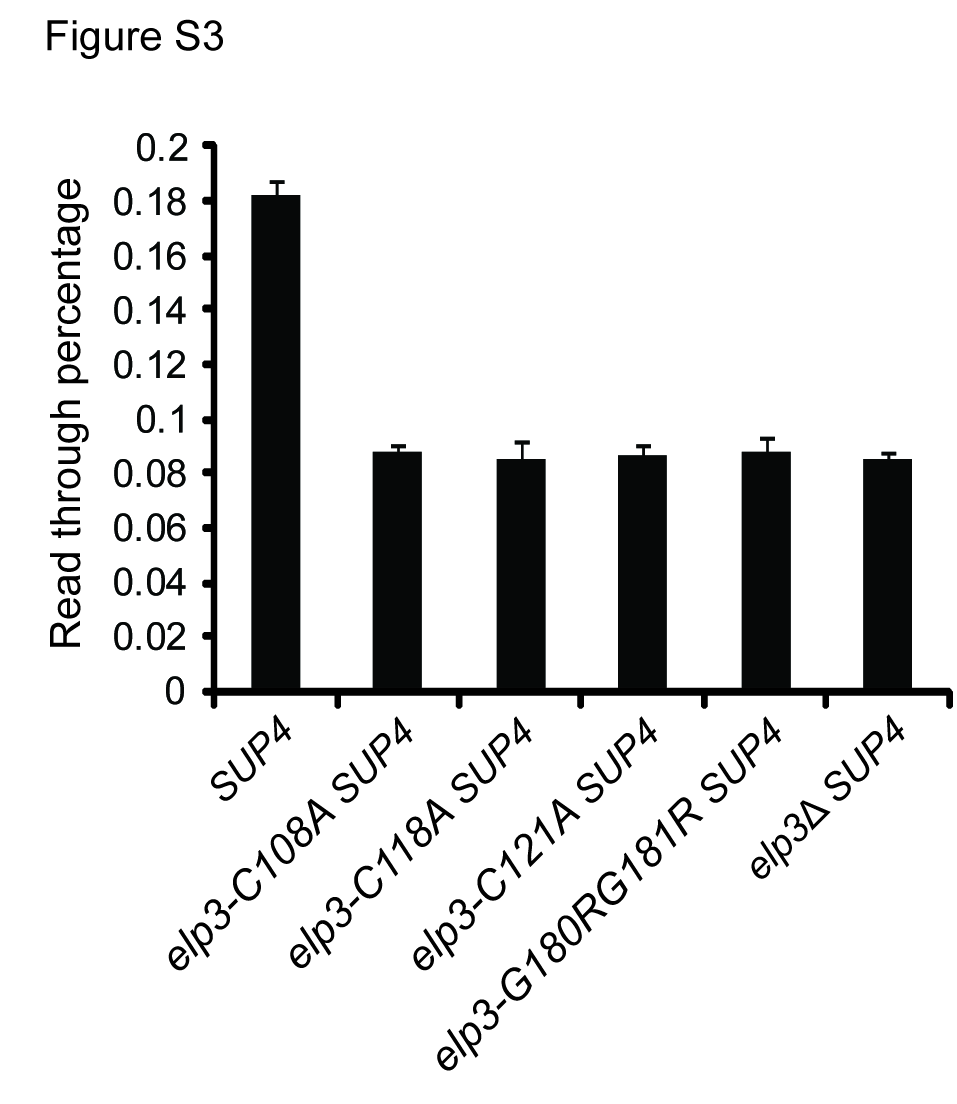

Supplement: Figure S3 — UAA stop codon read through by Sup4 tRNA in strains with different alleles of elp3. Read through levels of UAA stop codon in SUP4 (UMY2894), elp3-C108A SUP4 (UMY3315), elp3-C118A SUP4 (UMY3316), elp3-C121A SUP4 (UMY3317), elp3-G180R G181R SUP4 (UMY3795) and elp3Δ SUP4 (UMY2915). Values were based on three independent experiments. The error bars represent the standard deviation. The value of SUP4 (UMY2894) was arbitrarily set to 1 and the others were normalized to UMY2894. The dual luciferase reporter system used for UAA stop codon read through [27] is described in Figure 6A. (TIF) [file pgen.1002258.s003.tif]
