# Supplementary material for: Elongator Complex Influences Telomeric Gene Silencing and DNA Damage Response by Its Role in Wobble Uridine tRNA Modification
Source: PLoS Genet. 2011 Sep 1;7(9):e1002258. doi: 10.1371/journal.pgen.1002258 (PMC3164696; doi:10.1371/journal.pgen.1002258)
Supplement: Table S1 — Yeast strains used in this study (see also [10], [12], [15], [43]). (DOC) [file pgen.1002258.s004.doc]

Table S1. Yeast strains used in this study

| Strain | Genotype | Source or reference |
| --- | --- | --- |
| W303-1A | *MATa leu2-3,112 trp1-1 can1-100 ura3-1 ade2-1 his3-11,15* | [43] |
| W303-1B | *MATα leu2-3,112 trp1-1 can1-100 ura3-1 ade2-1 his3-11,15* | [43] |
| UMY3104 | *leu2-3,112/leu2-3,112 trp1-1/trp1-1 ura3-1/ura3-1 ade2-1/ade2-1 his3-11,15/his3-11,15 can1-100/can1-100* | [12] |
| UMY2843 | *MATα leu2-3,112 trp1-1 can1-100 ura3-1 ade2-1 his3-11,15, elp3::KanMX4* | [10] |
| UMY3783 | *MATa leu2-3,112 trp1-1 can1-100 ura3-1 ade2-1 his3-11,15, elp1::KanMX4* | This study |
| UMY3784 | *MATa leu2-3,112 trp1-1 can1-100 ura3-1 ade2-1 his3-11,15, elp2::KanMX4* | This study |
| UMY3269 | *MATa leu2-3,112 trp1-1 can1-100 ura3-1 ade2-1 his3-11,15, elp3::KanMX4* | This study |
| UMY3785 | *MATa leu2-3,112 trp1-1 can1-100 ura3-1 ade2-1 his3-11,15, elp4::KanMX4* | This study |
| UMY3786 | *MATa leu2-3,112 trp1-1 can1-100 ura3-1 ade2-1 his3-11,15, elp5::KanMX6* | This study |
| UMY3787 | *MATa leu2-3,112 trp1-1 can1-100 ura3-1 ade2-1 his3-11,15, elp6::KanMX4* | This study |
| UMY2584 | *MATa leu2-3,112 trp1-1 can1-100 ura3-1 ade2-1 his3-11,15, TELVIIL::URA3, TELVR::ADE2* | Jasper Rine |
| UMY3788 | *MATa leu2-3,112 trp1-1 can1-100 ura3-1 ade2-1 his3-11,15, TELVIIL::URA3, TELVR::ADE2, elp1::KanMX4* | This study |
| UMY3789 | *MATa leu2-3,112 trp1-1 can1-100 ura3-1 ade2-1 his3-11,15, TELVIIL::URA3, TELVR::ADE2, elp2::KanMX4* | This study |
| UMY3790 | *MATa leu2-3,112 trp1-1 can1-100 ura3-1 ade2-1 his3-11,15, TELVIIL::URA3, TELVR::ADE2, elp3::KanMX4* | This study |
| UMY3791 | *MATa leu2-3,112 trp1-1 can1-100 ura3-1 ade2-1 his3-11,15, TELVIIL::URA3, TELVR::ADE2, elp4::KanMX4* | This study |
| UMY3792 | *MATa leu2-3,112 trp1-1 can1-100 ura3-1 ade2-1 his3-11,15, TELVIIL::URA3, TELVR::ADE2, elp5::KanMX6* | This study |
| UMY3793 | *MATa leu2-3,112 trp1-1 can1-100 ura3-1 ade2-1 his3-11,15, TELVIIL::URA3, TELVR::ADE2, elp6::KanMX4* | This study |
| UMY2893 | *MATα leu2-3,112 trp1-1 can1-100 ura3-1 ade2-1 his3-11,15, SUP4* | [10] |
| UMY2894 | *MATa leu2-3,112 trp1-1 can1-100 ura3-1 ade2-1 his3-11,15, SUP4* | [10] |
| UMY2915 | *MATa leu2-3,112 trp1-1 can1-100 ura3-1 ade2-1 his3-11,15, SUP4, elp3::KanMX4* | [10] |
| UMY3314 | *MATα leu2-3,112 trp1-1 can1-100 ura3-1 ade2-1 his3-11,15, SUP4, elp3-C103A* | This study |
| UMY3315 | *MATα leu2-3,112 trp1-1 can1-100 ura3-1 ade2-1 his3-11,15, SUP4, elp3-C108A* | This study |
| UMY3316 | *MATα leu2-3,112 trp1-1 can1-100 ura3-1 ade2-1 his3-11,15, SUP4, elp3-C118A* | This study |
| UMY3317 | *MATα leu2-3,112 trp1-1 can1-100 ura3-1 ade2-1 his3-11,15, SUP4, elp3-C121A* | This study |
| UMY3794 | *MATa leu2-3,112 trp1-1 can1-100 ura3-1 ade2-1 his3-11,15, SUP4, elp3-G168R* | This study |
| UMY3795 | *MATa leu2-3,112 trp1-1 can1-100 ura3-1 ade2-1 his3-11,15, SUP4, elp3-G180R G181R* | This study |
| UMY3060 | *MATa leu2-3,112 trp1-1 can1-100 ura3-1 ade2-1 his3-11,15, SUP4, elp3-Y540A* | [10] |
| UMY3061 | *MATa leu2-3,112 trp1-1 can1-100 ura3-1 ade2-1 his3-11,15, SUP4, elp3-Y541A* | [10] |
| UMY3442 | *MATa leu2-3,112 trp1-1 can1-100 ura3-1 ade2-1 his3-11,15, tuc2::KanMX4* | [15] |
| UMY3804 | *MATa leu2-3,112 trp1-1 can1-100 ura3-1 ade2-1 his3-11,15, TELVIIL::URA3, TELVR::ADE2, tuc2::KanMX4* | This study |
| UMY3798 | *MATa leu2-3,112 trp1-1 can1-100 ura3-1 ade2-1 his3-11,15, rtt109::KanMX4* | This study |
| UMY3800 | *MATa leu2-3,112 trp1-1 can1-100 ura3-1 ade2-1 his3-11,15, asf1::KanMX4* | This study |
| UMY3805 | *MATa leu2-3,112 trp1-1 can1-100 ura3-1 ade2-1 his3-11,15, asf1::KanMX4, elp3::KanMX4* | This study |
| UMY3807 | *MATa leu2-3,112 trp1-1 can1-100 ura3-1 ade2-1 his3-11,15, rtt109::KanMX4, elp3::KanMX4* | This study |
| UMY2329 | *MATa ade2::hisG can1::hisG his3-11 leu2 trp1Del ura3-52 TelVR::ADE2 SIR4-13Myc-KanMX* | Susan Gasser |
| UMY3844 | *MATα ade2::hisG can1::hisG his3-11 leu2 trp1Del ura3-52 elp3::KanMX SIR4-13Myc-KanMX* | This study |
