# Supplementary material for: Elongator Complex Influences Telomeric Gene Silencing and DNA Damage Response by Its Role in Wobble Uridine tRNA Modification
Source: PLoS Genet. 2011 Sep 1;7(9):e1002258. doi: 10.1371/journal.pgen.1002258 (PMC3164696; doi:10.1371/journal.pgen.1002258)
Supplement: Table S2 — Plasmids used in this study (see also [10]–[11], [27], [44]–[45]). (DOC) [file pgen.1002258.s005.doc]

Table S2. Plasmids used in this study

| Plasmids | Discription | Source or reference |
| --- | --- | --- |
| pRS315 | YCp, *LEU2* | [44] |
| pABY1514 | pRS315-*ELP3* | [10] |
| pABY1986 | pRS315-*elp3-C103A* | This study |
| pABY1987 | pRS315-*elp3-C108A* | This study |
| pABY1988 | pRS315-*elp3-C118A* | This study |
| pABY1989 | pRS315-*elp3-C121A* | This study |
| pABY1990 | pRS315-*elp3-G168R* | This study |
| pABY1991 | pRS315-*elp3-G180RG181R* | This study |
| pABY1992 | pRS315-*elp3-Y540A* | This study |
| pABY1993 | pRS315-*elp3-Y541A* | This study |
| pABY1554 | pRS306-*ELP3* | [10] |
| pABY1672 | pRS306-*elp3-C103A* | This study |
| pABY1673 | pRS306-*elp3-C108A* | This study |
| pABY1676 | pRS306-*elp3-C118A* | This study |
| pABY1677 | pRS306-*elp3-C121A* | This study |
| pABY1984 | pRS306-*elp3-G168R* | This study |
| pABY1985 | pRS306-*elp3-G180RG181R* | This study |
| pABY1559 | pRS306-*elp3-Y540A* | [10] |
| pABY1560 | pRS306-*elp3-Y541A* | [10] |
| pRS425 | YEp, *LEU2* | [45] |
| pABY1653 | pRS425-*tK(UUU)-tQ(UUG)-tE(UUC)* | [11] |
| pABY1707 | pRS425-*tK(UUU)-tQ(UUG)* | [11] |
| pABY1649 | pRS425-*tK(UUU)-tE(UUC)* | [11] |
| pABY1661 | pRS425-*tQ(UUG)-tE(UUC)* | [11] |
| pABY1604 | pRS425-*tK(UUU)* | [11] |
| pABY1499 | pRS425-*tQ(UUG)* | [11] |
| pABY1479 | pRS425-*tE(UUC)* | [11] |
| pDB722 | Dual luciferase reporter construct with codon CAA C | [27] |
| pDB723 | Dual luciferase reporter construct with codon UAA C | [27] |
| pRS424 | YEp, *TRP1* | [45] |
| pABY1975 | pRS424-*SIR4* | This study |
